# Genome-wide association study and genome prediction of tallness trait in spinach phenotyping

**DOI:** 10.3389/fpls.2025.1654904

**Published:** 2025-09-29

**Authors:** Ibtisam Alatawi, Haizheng Xiong, Hanan Alkabkabi, Kenani Chiwina, Beiquan Mou, Qun Luo, Yuejun Qu, Renjie Du, Awais Riaz, Derrick J. Harrison, Ainong Shi

**Affiliations:** ^1^ Department of Horticulture, University of Arkansas, Fayetteville, AR, United States; ^2^ Department of Biology, Faculty of Science, University of Tabuk, Tabuk, Saudi Arabia; ^3^ Wenzhou Academy of Agricultural Sciences, Wenzhou, Zhejiang, China; ^4^ Sam Farr United States Crop Improvement and Protection Research Center, U.S. Dept. of Agriculture, Agricultural Research Service (USDA-ARS), Salinas, CA, United States; ^5^ Department of Crop, Soil, and Environmental Sciences, University of Arkansas, Fayetteville, AR, United States

**Keywords:** genome-wide association study (GWAS), genomic prediction (GP), plant height, single-nucleotide polymorphism (SNP), *Spinacia oleracea* L., spinach, tallness

## Abstract

Plant height is a critical agronomic trait in spinach (*Spinacia oleracea* L.), influencing both mechanical harvesting efficiency and overall yield. In this study, plant height variation was evaluated in 307 United States Department of Agriculture (USDA) germplasm accessions, which were phenotyped and genotyped using 15,058 single-nucleotide polymorphisms (SNPs) obtained from whole-genome resequencing. A genome-wide association study (GWAS) was conducted using the General Linear Model (GLM), Mixed Linear Model (MLM), Multiple Loci Mixed Model (MLMM), Fixed and Random Model Circulating Probability Unification (FarmCPU), and Bayesian-information and Linkage-disequilibrium Iteratively Nested Keyway (BLINK) models implemented in the Genomic Association and Prediction Integrated Tool version 3 (GAPIT3). Ten SNPs were significantly associated with plant height: (i) SOVchr1_10780051 (10,780,051 bp) on chromosome (chr) 1; (ii) SOVchr2_68062488 (68,062,488 bp) on chr 2; (iii) SOVchr4_38323167 (38,323,167 bp), SOVchr4_188084317 (188,084,317 bp), and SOVchr4_188084338 (188,084,338 bp) on chr 4; (iv) SOVchr5_70192260 (70,192,260 bp) and SOVchr5_105368320 (105,368,320 bp) on chr 5; and (v) SOVchr6_8139833 (8,139,833 bp), SOVchr6_90951127 (90,951,127 bp), and SOVchr6_91175684 (91,175,684 bp) on chr 6. Genomic prediction (GP) models were applied to estimate genomic estimated breeding values (GEBV) for plant height, achieving an r-value of 0.55 using GWAS-derived SNP markers in cross-population prediction. The integration of GWAS and GP provides insights into the genetic architecture of plant height in spinach and supports marker-assisted breeding strategies to enhance crop management and economic returns.

## Introduction

Spinach (*Spinacia oleracea* L.) is a highly nutritious leafy vegetable, widely cultivated in the United States and globally (Shi et al., 2016; Tang et al., 2015). Its increasing demand is driven by consumer awareness of its rich nutritional profile, including essential vitamins, minerals, antioxidants, and bioactive compounds such as carotenoids and flavonoids (Frary et al., 2010; Rashid et al., 2020). Among its key agronomic traits, plant height plays a crucial role in spinach production and management (Jones et al., 2019). Taller spinach plants are likely easier for harvesting machinery to reach and cut cleanly, thereby reducing yield loss and improving throughput. For leafy vegetables harvested using horizontal or topper-style cutters, increased plant height can result in less missed crop and better compatibility with mechanical harvesting. Taller plants and a higher position of the first primary branch have been shown to significantly improve machine-harvest efficiency in green chile cultivars (Joukhadar et al., 2018). In legumes, the trait ‘height to first pod’ (HFP) is critical, as pods must be positioned above the cutterbar height to avoid harvest loss. Improved HFP correlates with reduced seed loss during mechanical harvesting (Kuzbakova et al., 2022). Height to first pod: A review of genetic and breeding approaches to improve combine harvesting in legume crops. Front Plant Sci. 13:948099. doi: 10.3389/fpls.2022.948099. However, optimizing plant height requires a balance, as taller plants must also resist lodging—a condition where plants collapse under adverse weather, leading to yield loss (Jones et al., 2019).

Plant height in spinach, like in other major crops such as rice and maize, is a polygenic trait governed by multiple genetic factors (Huang and Han, 2016). Traditional quantitative trait loci (QTL) mapping approaches have been useful in identifying large-effect loci but often fail to detect small-effect loci that collectively influence complex traits (Yu and Buckler, 2006). This limitation underscores the need for genome-wide approaches such as genome-wide association studies (GWAS) and genomic prediction (GP), which enable the identification of multiple loci contributing to plant height and improve breeding efficiency through genome-wide marker predictions.

The substantial phenotypic variation observed in spinach plant height reflects its rich genetic diversity, making it an excellent candidate for advanced genomic studies and breeding efforts (Huang and Han, 2016; Yu and Buckler, 2006). GWAS has been a powerful tool for dissecting complex traits by identifying associations between single-nucleotide polymorphism (SNP) and phenotypic variation. In spinach, GWAS has successfully identified genetic loci controlling plant height, downy mildew resistance, and leaf morphology (Cai et al., 2018). By leveraging high-density SNP markers, GWAS facilitates the discovery of key genetic regions associated with important traits, supporting marker-assisted selection (MAS) in breeding programs. For instance, previous studies have identified height-related SNPs on chromosomes 2 and 6, linked to increased plant tallness (Shi et al., 2016). The high resolution of GWAS enables the detection of both major and minor loci, enhancing genetic improvement strategies without compromising other critical traits such as leaf texture, flavor, or pest resistance (Jones et al., 2019). The integration of GWAS with traditional breeding methods can significantly improve selection efficiency (Korte and Farlow, 2013), supporting the development of spinach varieties optimized for both agricultural productivity and consumer preferences.

GS is an advanced breeding approach that utilizes genome-wide markers to predict genetic potential before trait expression (Goddard and Hayes, 2007). While GS has been successfully implemented in maize, wheat, and rice to enhance yield, disease resistance, and stress tolerance (Crossa et al., 2017), its application in spinach remains limited (Bhattarai and Shi, 2021). Nevertheless, studies in other crops highlight the potential of GS to accelerate breeding cycles and improve cultivar development (Gaynor et al., 2017). GS could be particularly valuable for optimizing plant height, biomass, and leaf morphology by enabling early selection of superior genotypes, reducing reliance on time-intensive field evaluations (Heffner et al., 2009). Expanding the application of GS in spinach breeding holds promise for improving agricultural efficiency and developing high-performing cultivars suited to market demands. GP as a GS parameter has been investigated in dozen of crops including spinach (Shi et al., 2021, 2022). Genomic estimated breeding values (GEBV) in GP is the key step in GS. Several approaches have been proposed for GEBV such as Best Linear Unbiased Prediction (BLUP) methods [(Genomic Best Linear Unbiased Prediction (gBLUP), Ridge Regression Best Linear Unbiased Prediction (RR-BLUP), Compressed Best Linear Unbiased Prediction (cBLUP), and Super Best Linear Unbiased Prediction (sBLUP)] and Bayesian methods (Bayes A (BA), Bayes B (BB), Bayes LASSO (BL), and Bayesian Ridge Regression (BRR) (Bhattarai et al., 2022a, b; Shi et al., 2021, 2022).

This study had two primary objectives: (1) to perform a GWAS to identify SNP markers associated with plant height in spinach, and (2) to implement GP models to assess the accuracy of these markers in predicting plant height. We utilized a dataset of 15,058 high-quality SNPs obtained from whole-genome resequencing of 307 USDA-GRIN spinach accessions, forming the basis for GWAS and GP analyses. Our findings contribute to a deeper understanding of the genetic architecture of plant height in spinach and provide valuable resources for breeding programs aimed at improving mechanical harvesting efficiency and overall crop performance.

## Materials and methods

### Plant material

A total of 307 spinach accessions were obtained from the United States Department of Agriculture (USDA) Germplasm Resources Information Network (GRIN) spinach germplasm repository. These accessions represented 30 countries, with the majority originating from Turkey (n = 96), the United States (n = 52), Afghanistan (n = 21), North Macedonia (n = 18), China (n = 16), Iran (n = 13), and Belgium (n = 11), collectively accounting for 74.9% of the total collection. Phenotypic assessments for plant height were conducted, and whole-genome resequencing was performed to generate genotypic data. Detailed information on these accessions is provided in **Supplementary Table S1**.

### Experimental design for plant height measurement

Phenotypic data for plant height were collected from the 307 accessions at the USDA Agricultural Research Service (ARS) research station in Salinas, CA (Chitwood et al., 2016). The experiment utilized pasteurized sandy loam soil in a greenhouse setting. Each accession was grown in plastic pots (10 × 10 × 10cm) filled with a 2:1 mixture of sand and soil (by volume). A randomized complete block design (RCBD) with three replications was implemented, with 10 plants per accession. Plant height was measured 55 days after planting as the distance from the soil surface to the highest leaf tip. Descriptive statistics, including mean, range, standard deviation (SD), and standard error (SE), were calculated using JMP Genomics v.17 (SAS Institute, Cary, NC). The trait distribution was visualized using Genomic Association and Prediction Integrated Tool version 3 (GAPIT v.3), and the mean plant height per accession was used for GWAS analysis.

### DNA extraction and whole-genome sequencing

Firstly, genomic DNA was extracted from freshly harvested leaves pooled from 5 to 10 plants per accession using the CTAB (hexadecyltrimethyl ammonium bromide) method. High-quality DNA was fragmented into 350-bp segments using a Covaris Ultrasonic Processor, and sequencing libraries were prepared following a standardized protocol (Van Dijk et al., 2014). Whole-genome resequencing (WGR) was performed using paired-end sequencing on the Illumina NovaSeq platform at approximately 10× genome coverage per sample, generating about 10 gigabases of sequence data per genotype. Sequencing was conducted by Beijing Genomics Institute (BGI) (https://www.bgi.com/). Approximately 6 million raw SNPs across 470 spinach accessions were initially identified by aligning the short reads to the Sp75 reference genome; this data was provided by BGI.

Secondly, these reads were re-aligned to the Monoe-Viroflay reference genome using the Texas A&M Bioinformatics Center pipeline. The Monoe-Viroflay spinach genome (Collins et al., 2019), obtained from SpinachBase (http://www.spinachbase.org/), was used as the reference genome. Alignment was performed using the Burrows-Wheeler Aligner (BWA v0.7.8-r455) (Li and Durbin, 2009). BAM (Binary Alignment/Map) files were sorted, and duplicate reads were removed using SAMtools (v0.1.19-44428cd) (Li et al., 2009). BAM files from the same sample were merged using the Picard toolkit (v1.111) (https://broadinstitute.github.io/picard/). SNP and InDel (insertion and deletion variant) calling was conducted using GATK (Genome Analysis Toolkit) (v3.5) (McKenna et al., 2010), yielding half a million raw SNPs from 470 spinach accessions provided by the Texas A&M Bioinformatics Center.

**Figure 1:**
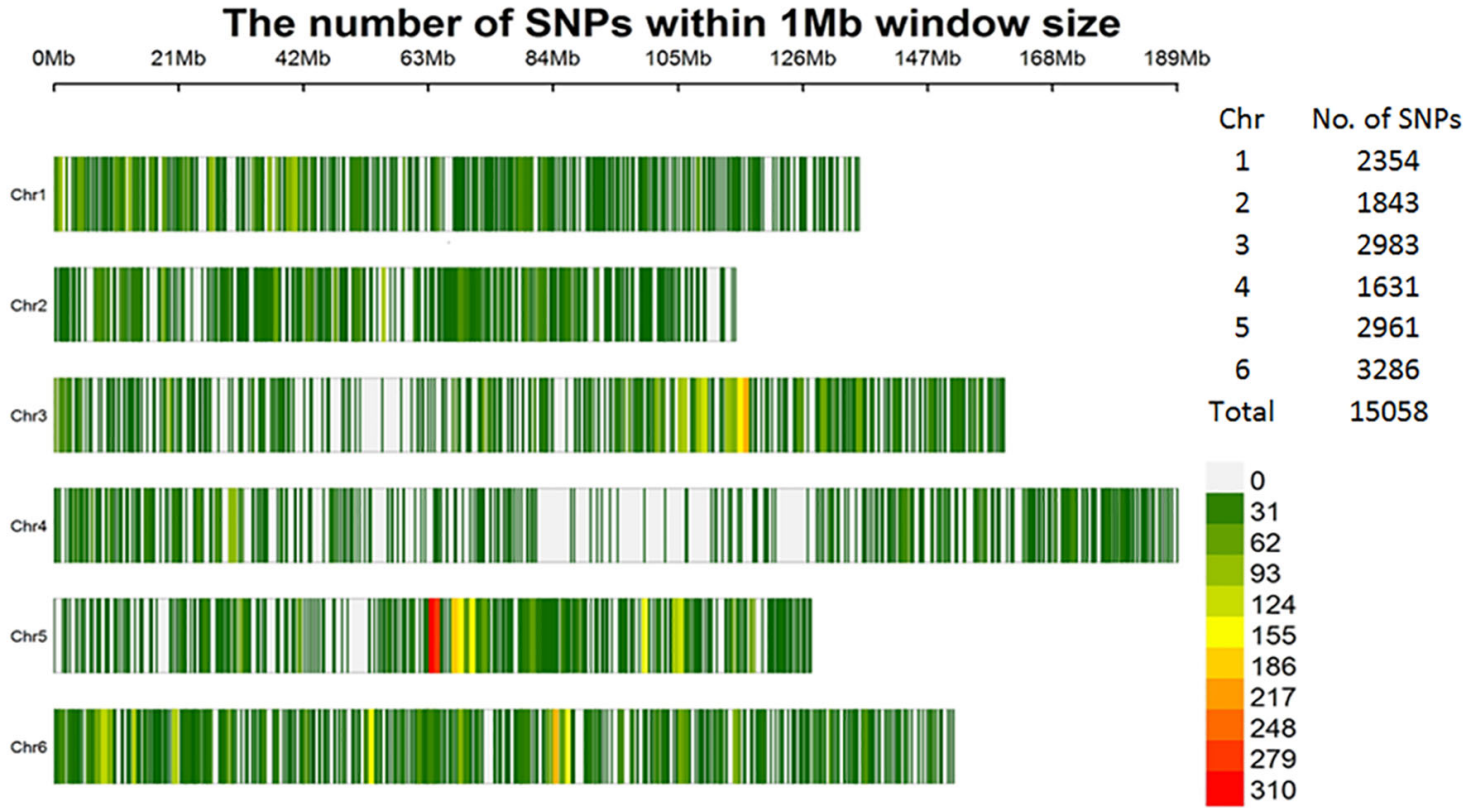
Distribution of the 15,058 high-quality SNPs within 1-Mb window sizes across six spinach chromosomes.

Thirdly, for the subset of 307 accessions used in this study, stringent filtering criteria were applied and keeping those SNPs with: minor allele frequency (MAF) > 5%, missing data rate < 7%, and heterozygosity rate < 15%. After filtering, 15,058 high-quality SNPs remained, distributed across the six spinach chromosomes (**Figure 1**). The SNP dataset has been published in the FigShare database and is accessible via the following link: https://doi.org/10.6084/m9.figshare.28603517.v1.

### Principal component analysis and genetic diversity

A model-based clustering method implemented in the STRUCTURE 2.3.4 program (Pritchard et al., 2000) was employed to infer the population structure of 307 spinach accessions based on 6,000 SNPs, with 1,000 SNPs randomly selected from each of the six spinach chromosomes. The burn-in period was set at 20,000 iterations, followed by 10,000 Markov Chain Monte Carlo iterations, using an admixture model with correlated allele frequencies independent for each run (Lv et al., 2012). Ten runs were performed for each simulated value of K, ranging from 1 to 10. The statistical value ΔK was calculated for each simulated K using the method of Evanno et al. (2005) to determine the optimal K representing the major population structure. Each spinach accession was subsequently assigned to a cluster (Q) based on the probability of membership estimated by the software, with a threshold probability of 0.50 or greater for assignment. Finally, a bar plot with “Sort by Q” was generated to visualize the population structure among spinach accessions at the optimal K.

Genetic diversity and principal component analysis (PCA) were also conducted using the GAPIT v. 3 (Wang and Zhang, 2021; https://zzlab.net/GAPIT/index.html). PCA was performed using eigenvalue decomposition with component numbers ranging from 2 to 10. A neighbor-joining phylogenetic tree was constructed to assess genetic relationships among the accessions.

### Genome-wide association study

GWAS was conducted using five statistical models implemented in GAPIT 3: the generalized linear model (GLM), mixed linear model (MLM), multiple loci mixed model (MLMM), Fixed and Random Model Circulating Probability Unification (FarmCPU) (Liu et al., 2016), and the Bayesian-information and Linkage-disequilibrium Iteratively Nested Keyway (BLINK) (Huang et al., 2019) model (Wang and Zhang, 2021; https://zzlab.net/GAPIT/index.html). Association significance was determined using a Bonferroni-corrected threshold (0.05/total SNPs), corresponding to a logarithm of odds (LOD) score of 5.48.

### Candidate gene identification

In this study, linkage disequilibrium (LD) with genetic distance (cM) between SNP loci was evaluated using Haploview (Barrett et al., 2005). Pairwise LD between SNPs was calculated as the squared allele-frequency correlation (r²) using TASSEL 5 (Bradbury et al., 2007). LD decay rates were estimated using 15,058 high-quality SNP markers across 307 accessions in two ways: (1) for each of the six chromosomes, as previously described (Zhou et al., 2015), and (2) for specific regions surrounding associated SNP markers, calculated by plotting r² values against physical distance (bp). The LD decay rate of the population was defined as the chromosomal distance at which the average r² declined to half of its maximum value (Kim et al., 2007; Lam et al., 2010).

Candidate genes near significant SNPs were identified based on the LD decay rate at each GWAS-identified SNP marker. When the LD decay rate could not be reliably estimated for a marker region, the chromosome-specific LD decay was used instead. LD heatmaps for candidate genes were generated using Haploview (Barrett et al., 2005) with Monoe-Viroflay genome annotations. Genome annotation data were accessed through SpinachBase (http://www.spinachbase.org/) or via FTP (http://spinachbase.org/ftp/genome/Monoe-Viroflay/).

### Genomic prediction for plant height

GP was performed using several models implemented in R packages. RR-BLUP was conducted using the ‘rrBLUP’ package (Endelman, 2011). Four Bayesian models—BA, BB, BL, and BRR—were implemented using the ‘BGLR’ package (Barili et al., 2018; Legarra et al., 2011). Additionally, Random Forest (RF) was applied using the ‘randomForest’ package (Ogutu et al., 2011), and Support Vector Machines (SVM) were implemented using the ‘kernlab’ package (Maenhout et al., 2007). These approaches have been previously utilized in GS studies (Ravelombola et al., 2019, 2020, 2021; Shi et al., 2021, 2022).

### Genomic prediction using different SNP sets

We examined ten randomly selected subsets of SNPs, ranging from 6 to 15,058 SNPs, designated as r6, r50, r100, r200, r500, r1000, r2000, r5000, r10000, and all.15,058SNPs. Additionally, four GWAS-derived SNP sets (m10: 10 markers; m2: 2 markers; m6_2pca: 6 markers with PCA=2; m6_3pca: 6 markers with PCA=3) were derived from a GWAS conducted on a panel of 307 accessions using five models—GLM, MLM, MLMM, FarmCPU, and BLINK—implemented in GAPIT3.​ GEBVs were calculated for each of the ten SNP sets (ten randomly selected SNP sets plus four GWAS derived marker sets) across all seven GP models (BA, BB, BL, BRR, rrBLUP, RF, and SVM). Each combination underwent 100 iterations, and the mean correlation coefficients (r-values) along with standard errors (SE) were computed to assess model performance. Boxplots illustrating the performance of GP models across different SNP sets were generated using the ‘ggplot2’ package in R (Wickham, 2016).

### GP by GWAS-derived SNP markers

#### GWAS-derived SNP markers from the whole panel and cross-population prediction

First, GWAS was conducted using five models (GLM, MLM, MLMM, FarmCPU, and BLINK), and the associated SNP markers were identified from these models in the entire GWAS panel (307 spinach accessions). Secondly, GP was performed using the GWAS-derived SNP markers to perform cross-population prediction analysis with five-fold cross-validation (training:validation = 4:1) using seven genomic prediction (GP) models: BA, BB, BL, BRR, rrBLUP, RF, and SVM.

#### GWAS-derived SNP markers from 80% of the whole panel

Both cross- and across-population predictions were performed for tallness using GWAS-derived associated SNP markers. The entire panel (307 accessions) was divided into two subsets: 80% as the training population (TP) (246 accessions) and 20% as the validation population (VP) (61 accessions). GWAS was performed on the 246 accessions using the GLM, MLM, FarmCPU, and BLINK models in GAPIT3. Associated SNPs with a LOD score (-log(P)) > 4.0 were selected from the four models and used to run the GP model 100 times, calculating GEBVs and estimating the average r-value each time. This process was repeated five times, and the mean r-value across the five replications was obtained as the prediction accuracy (average r-value). Three GP types were tested: ‘Across-prediction’, ‘Cross-prediction’, and ‘Cross_self.prediction’.

- Across_prediction uses GWAS-derived SNP markers from the training set (80% of the population, 246 accessions) to predict the validation set (20% – 61 accessions).
- Cross_prediction uses all associated SNP markers from the five repeats to predict the entire population (307 accessions).
- Cross_self.prediction uses GWAS-derived SNP markers from the training set (80% of the population) to predict itself.

Additionally, GP was performed with five GP models (RF, BA, BB, BL, and BRR), and GEBVs were calculated for all models. Each replication in each model was run 100 times, and mean r-values along with SE were computed. Boxplots illustrating GP model performance across SNP sets were generated using ggplot2 in R.

#### GWAS-derived SNP markers using GAGBLUP in GAPIT3

GP was conducted using the GAGBLUP (BLINK) model in GAPIT3 on the entire population of 307 accessions, referred to as the reference prediction (cross_self.prediction), where the 307 accessions were used as both the training population (TP) and validation population (VP). Additionally, following the same approach as described above, the entire panel (307 accessions) was divided into two subsets: 80% as the TP (246 accessions) and 20% as the VP (61 accessions). GWAS was performed using the BLINK model only in GAPIT3, and the associated SNPs with a LOD score (-log(P)) > 5.48 were selected to run the GAGBLUP model in GAPIT3. Both across- and cross-population predictions were performed. The across-population prediction (Across-prediction) was performed using the associated SNP markers from the TP (246 accessions) to predict the GEBVs in the VP (61 accessions). Cross-population prediction was performed using the associated SNP markers from the TP (246 accessions) to predict the GEBVs in the TP itself (246 accessions).

## Results

### Phenotyping of tallness

**Figure 2:**
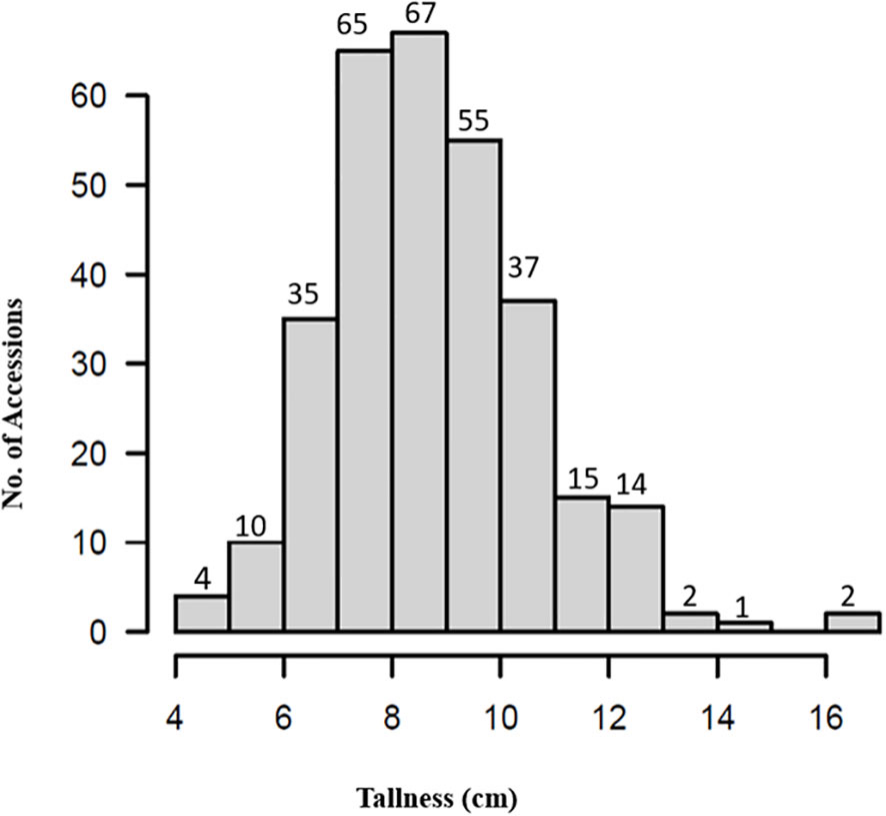
Distribution of plant tallness in the 307 spinach accessions.

Phenotypic data for plant height (tallness) across the 307 spinach accessions (**Supplementary Table S1**) exhibited a near-normal distribution (**Figure 2**), with heights ranging from 4.5 to 16.2cm. The shortest accession, PI 303138, measured 4.5cm, while the tallest, PI 177557, reached 16.2cm, approximately 11.7cm taller (**Supplementary Figures S1A–C**). The mean plant height was 8.8cm “standard deviation (SD) = 1.9”, with a coefficient of variation of 21.3%. The observed variation in plant height demonstrates the suitability of this panel for GWAS.

Seven accessions—PI 445784, PI 192945, PI 664497, PI 478393, PI 177558, and PI 433209—were identified as exceptionally tall, each exceeding 13cm in height (**Supplementary Figures S1E–G**). These accessions represent valuable genetic resources for breeding programs aimed at enhancing plant height in spinach.

### PCA and phylogenetic analysis

**Figure 3:**
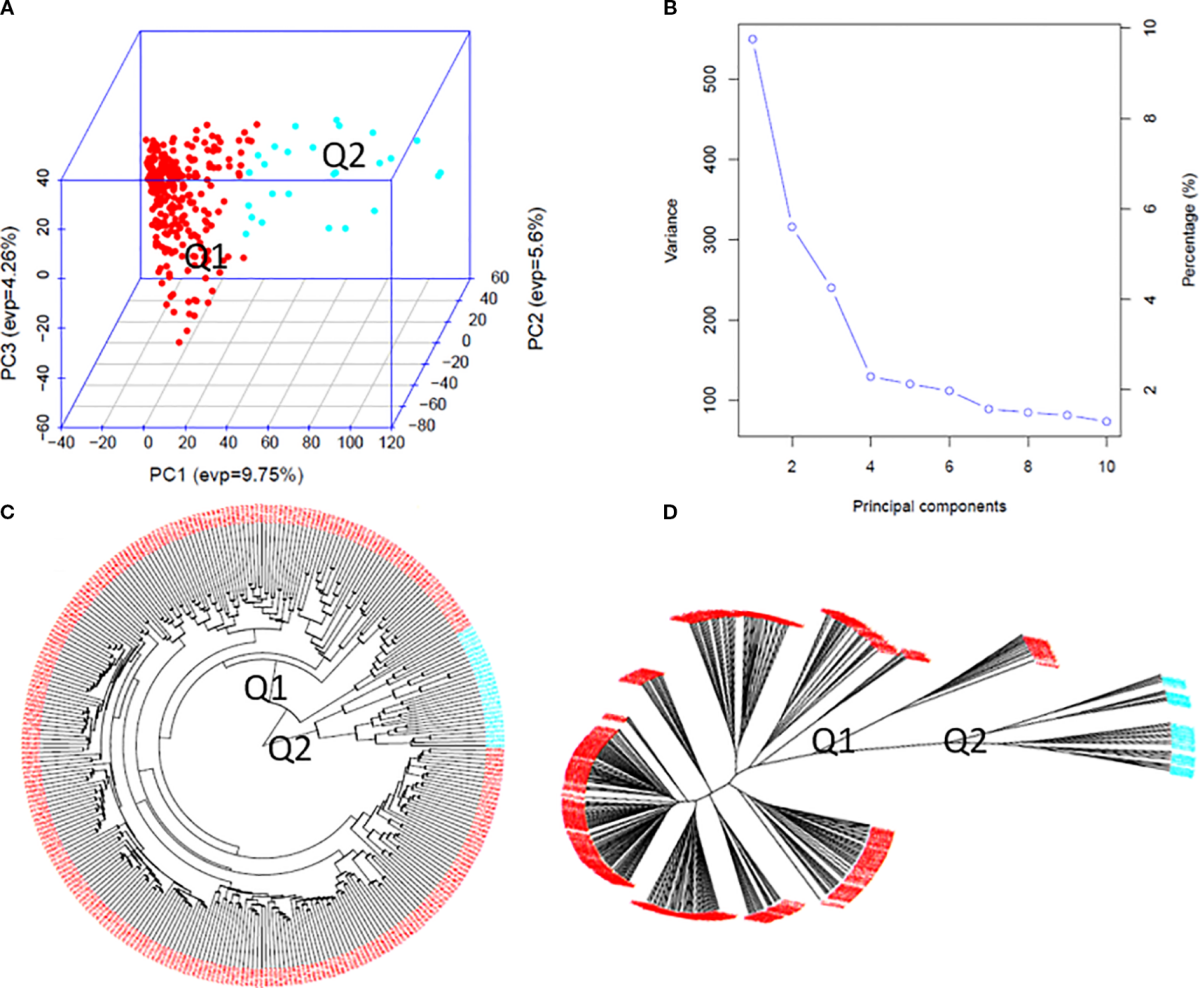
Population genetic diversity analysis in the association panel consisted of 307 USDA spinach germplasm accessions. **(A)** 3D graphical plot of the principal component analysis (PCA), **(B)** PCA.eigenValue plot drawn by GAPIT 3, and Phylogenetic trees [**(C)** fan and **(D)** unrooted] drawn by neighbor-joining method in two sub-populations.

Population structure analysis of the 307 spinach accessions revealed two major clusters (Q1 and Q2) based on GAPIT3 and STRUCTURE 2.3.4. A peak in Delta K values from STRUCTURE (**Supplementary Figure S2a-A**) confirmed at least two distinct genetic groups. GAPIT3 results are shown in a 3D PCA plot (**Figure 3A**), PCA eigenvalue plot (**Figure 3B**), and phylogenetic trees (**Figures 3C, D**). A secondary peak in Delta K (**Supplementary Figure S2a-B**) suggested three subpopulations (Q1, Q2, Q3, plus a mixed group). The corresponding PCA and phylogenetic results are presented in **Supplementary Figures S2b (A–D)**, while detailed two-ring phylogenetic trees for all accessions are shown in **Supplementary Figures S2a (C, D)**. Both two-subpopulation (Q=2) and three-subpopulation (Q=3) models were therefore applied in GWAS to identify SNPs associated with tallness.

### Association study

**Figure 4:**
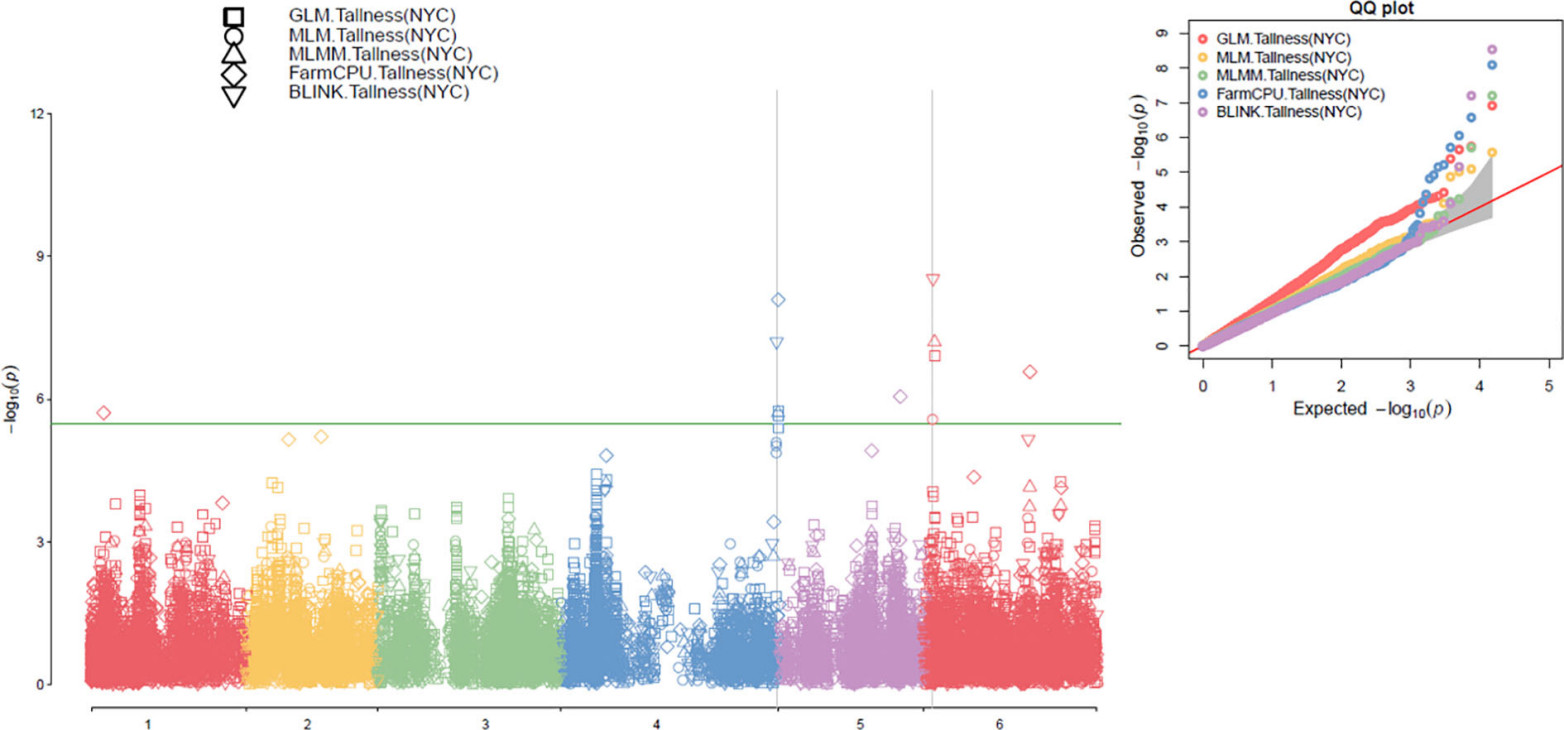
Multiple Manhattan plots (left) and QQ plots (right) generated using GLM, MLM, MLMM, FarmCPU, and BLINK models in GAPIT3 for the tallness trait in 307 spinach accessions with PCA=2.

In this study, association analyses for plant height (tallness) were performed using five models—GLM, MLM, MLMM, FarmCPU, and BLINK—in GAPIT3 with PCA set to 2 and 3. QQ plots comparing observed and expected LOD (−log10(P-value)) distributions showed significant deviations, which were consistent across multiple models in the 307 spinach accessions (**Figure 4** right, **Supplementary Figure S3** right). These results indicate the presence of SNP associations with plant height in the analyzed population.

The association analysis results for the tallness trait were visualized in Manhattan plots (**Figure 4**, left; **Supplementary Figure S3**, left) using five models implemented in GAPIT3: GLM, MLM, MLMM, FarmCPU, and BLINK. In the plots, each SNP is represented as a point, with chromosomal positions shown on the x-axis and –log10(P-value) on the y-axis. SNPs with LOD values greater than the significance threshold of 5.48 were considered significantly associated with the tallness trait. Across the five models, ten SNPs were identified as significantly associated with the tallness trait, each exceeding the threshold in at least one model under both runs with PCA=2 and PCA=3 (**Table 1**).

**Table 1:** Ten SNP markers associated with the tallness trait in spinach, identified using five models in GAPIT3 (GLM, MLM, MLMM, FarmCPU, and BLINK) with both PCA=2 and PCA=3.

SNP	Chr	Pos	MAF%	LOD = [-log10(P-value)]						Allele (Short)	Allele (Tall)	PVE (%) (Model)	Model (LOD>5.48)	Q-matrix (PCA)
				GAPIT3					*t*-test					
				BLINK	FarmCPU	MLMM	GLM	MLM						
SOVchr1_10780051	1	10780051	10.7	2.28	5.71	2.29	2.72	2.74	1.80	G	A	5.06(FarmCPU)	farmcpu	PCA=2
SOVchr2_68062488	2	68062488	11.2	3.35	5.67	2.88	3.51	2.97	1.09	A	G		FarmCPU	PCA=3
SOVchr4_38323167	4	38323167	10.9	5.57	6.47	4.68	4.63	4.30	2.09	A	G	5.45	FarmCPU,BLINK	PCA=3
SOVchr4_188084317	4	188084317	12.5	7.20	8.09	0.39	5.02	5.74	3.59	T	G	24.57 (blink) 29.15(Farmcpu)	blink.farmcpu.glm	PCA=2
SOVchr4_188084338	4	188084338	11.1	5.74	5.28	5.24	4.94	4.85	2.55	T	C	8.00(blin)	BLINK	PCA=3
				0.49	0.25	5.71	5.09	5.65				24.42(mlmm,glm)	mlmm.glm(mlm=5.09)	PCA=2
SOVchr5_70192260	5	70192260	15.6	2.04	5.53	1.12	1.08	1.50	1.31	C	A		FarmCPU	PCA=3
SOVchr5_105368320	5	105368320	11.9	2.45	6.05	2.52	2.69	2.36	1.97	A	G	6.54(farmcpu)	farmcpu	PCA=2
SOVchr6_8139833	6	8139833	12.7	7.64	3.43	6.56	6.10	5.47	5.19	G	T	6.11(blink) 25.42(glm)	BLINK,GLM	PCA=3
				8.53	2.73	7.20	5.57	6.92				20.43 (blink) 20.52(mlmm,glm) 26.91(mlm)	blink.mlmm. mlm.glm	PCA=2
SOVchr6_90951127	6	90951127	16.6	1.08	6.57	3.74	2.98	2.90	2.14	A	C	6.74(farmcpu)	farmcpu	PCA=2
SOVchr6_91175684	6	91175684	12.4	5.60	6.04	4.31	3.90	3.76	2.12	G	C	4.07(blink)	FarmCPU,BLINK	PCA=3
				5.16	0.13	4.15	3.50	3.30					blink=5.16	PCA=2

Results from a t-test and the percentage of phenotypic variance explained (PVE%) are also presented.

Notably, SOVchr6_8139833 consistently exhibited a LOD value greater than 5.48 across three models (BLINK, GLM, and MLMM) under both PCA=2 and PCA=3. It also showed LOD values of 6.92 (MLM, PCA=2) and 5.47 (MLM, PCA=3), along with high PVE values of up to 26.91% in MLMM (PCA=2) and 25.42% in GLM (PCA=3), indicating a strong and stable association. In contrast, lower LOD values of 3.43 (PCA=3) and 2.73 (PCA=2) were observed in the FarmCPU model (**Table 1**). Similarly, SOVchr4_188084338 was strongly associated in the BLINK model (LOD=5.74), while the other models reported moderate LOD values (> 4.85) when PCA=3. This SNP also exceeded the significance threshold in MLMM (LOD=5.71) and MLM (LOD=5.85), but showed lower values in GLM (LOD=4.94) and very weak signals in BLINK and FarmCPU (< 0.5) (**Table 1**), suggesting an association with tallness that is less consistent across models. Additional significant associations were detected for SOVchr4_38323167 and SOVchr4_188084317 on chromosome 4, as well as SOVchr6_90951127 and SOVchr6_91175684 on chromosome 6, highlighting their potential roles in the genetic regulation of plant height in spinach. Furthermore, SOVchr1_10780051, SOVchr2_68062488, SOVchr5_70192260, and SOVchr5_105368320 were significantly associated in the FarmCPU model, each exceeding the LOD threshold of 5.48 (**Table 1**). Collectively, the identification of these ten SNPs, particularly those surpassing the stringent threshold on chromosomes 1, 2, 4, 5, and 6, underscores their importance as genetic markers linked to tallness. These findings provide valuable insights into the genetic architecture of plant height in spinach and offer promising targets for marker-assisted breeding. The distribution of the ten associated SNP markers among the 307 spinach accessions revealed distinct phenotypic differences in plant height across allele combinations (**Supplementary Figure S4**), further reinforcing their relevance to this trait.

### Candidate gene identification/detection

LD decay analysis revealed rates of 170 kb, 140 kb, 330 kb, 50 kb, 210 kb, and 160 kb for chromosomes 1, 2, 3, 4, 5, and 6, respectively (**Supplementary Figure S5A**). The LD decay of the ten SNP markers associated with tallness ranged from 10 kb to 100 kb (**Supplementary Figure S5B**). For three SNPs on chromosome 4 (SOVchr4_38323167, SOVchr4_188084317, and SOVchr4_188084338), LD decay could not be reliably estimated; therefore, all genes within 50 kb (chromosome 4’s LD decay) were included. In total, 33 genes located within the LD regions of the ten associated SNPs are listed in **Supplementary Table S2**.

Based on proximity to associated SNP markers, nine genes were identified as candidate genes for tallness (**Table 2**). These include:

**Table 2:** Nine candidate genes identified within the specific LD decay regions corresponding to eight of the ten associated SNP markers (listed in **Table 1**) for the tallness trait in spinach.

Gene	Chr	Start_pos (bp)	End_pos (bp)	Gene size (bp)	Annotation_gene_name	SNP	Chr	Pos (bp)	From gene start (bp)	From gene end (bp)	Comment
*SOV1g002210*	1	10770653	10770964	311	RNase H domain-containing protein	SOVchr1_10780051	1	10780051	9398	9087	<10kb
*SOV2g015180*	2	68078694	68081726	3033	CCHC-type domain-containing protein	SOVchr2_68062488	2	68062488	-16206	-19238	<17kb
*SOV4g016060*	4	38326318	38334619	8302	U6 snRNA-associated Sm-like protein LSm5	SOVchr4_38323167	4	38323167	-3151	-11452	<4kb
*SOV4g059190*	4	188080640	188082495	1856	outer envelope membrane protein 7-like	SOVchr4_188084338	4	188084338	3698	1843	<2kb
						SOVchr4_188084317	4	188084317	3677	1822	<2kb
*SOV4g059200*	4	188085284	188087037	1754	Epimerase domain-containing protein	SOVchr4_188084338	4	188084338	-946	-2699	<1kb
						SOVchr4_188084317	4	188084317	-967	-2720	<1kb
*SOV5g028680*	5	70188248	70192130	3883	Cleavage and polyadenylation specificity factor subunit 2	SOVchr5_70192260	5	70192260	4012	130	130bp
*SOV6g002670*	6	8260666	8265472	4807	F-box domain-containing protein	SOVchr6_8139833	6	8139833	-120833	-125639	<121kb
*SOV6g002680*	6	8266176	8267578	1403	F-box domain-containing protein				-126343	-127745	<127kb
*SOV6g020520*	6	91176079	91179277	3198	LETM1 and EF-hand domain-containing protein 1 mitochondrial	SOVchr6_91175684	6	91175684	-395	-3593	395bp

- SOV1g002210 (RNase H domain-containing protein), located at 10,770,653–10,770,964 bp on chromosome 1, <10 kb from SNP SOVchr1_10780051. RNase H domain-containing proteins, such as Rht8 in wheat, regulate plant height through gibberellin (GA) biosynthesis, modulating stem elongation and contributing to semi-dwarf phenotypes (Zhou et al., 2023).
- SOV2g015180 (CCHC-type domain-containing protein), at 68,078,694–68,081,726 bp on chromosome 2, <17 kb from SNP SOVchr2_68062488. CCHC-type zinc finger proteins (CCHC-ZFPs) are involved in plant growth, development, and environmental adaptation (Sun et al., 2022).
- SOV4g016060 (U6 snRNA-associated Sm-like protein LSm5), at 38,326,318–38,334,619 bp on chromosome 4, near SNP SOVchr4_38323167.
- SOV4g059190 (outer envelope membrane protein 7-like) and SOV4g059200 (epimerase domain-containing protein), at 188,080,640–188,082,495 bp and 188,085,284–188,087,037 bp on chromosome 4, near SNP SOVchr4_188084338.
- SOV6g002670 and SOV6g002680 (F-box domain-containing proteins), at 8,260,666–8,265,472 bp and 8,266,176–8,267,578 bp on chromosome 6, ~121–127 kb from SNP SOVchr6_8139833. F-box proteins regulate plant height via the ubiquitin–proteasome system, modulating hormone signaling and stem elongation (Hua et al., 2020; Xu et al., 2021).
- SOV5g028680 (cleavage and polyadenylation specificity factor subunit 2) on chromosome 5, 70,188,248–70,192,130 bp, near SNP SOVchr5_70192260.
- SOV6g020520 (LETM1 and EF-hand domain-containing protein 1, mitochondrial) on chromosome 6, 91,176,079–91,179,277 bp, near SNP SOVchr6_91175684.

LD heatmaps of the regions surrounding these nine candidate genes (**Supplementary Figure S6**) showed that no SNPs were located within the genes or in the same LD regions, highlighting their potential regulatory roles in tallness.

### Genomic prediction for genomic selection of tallness trait

#### Genomic prediction using different SNP sets

**Figure 5:**
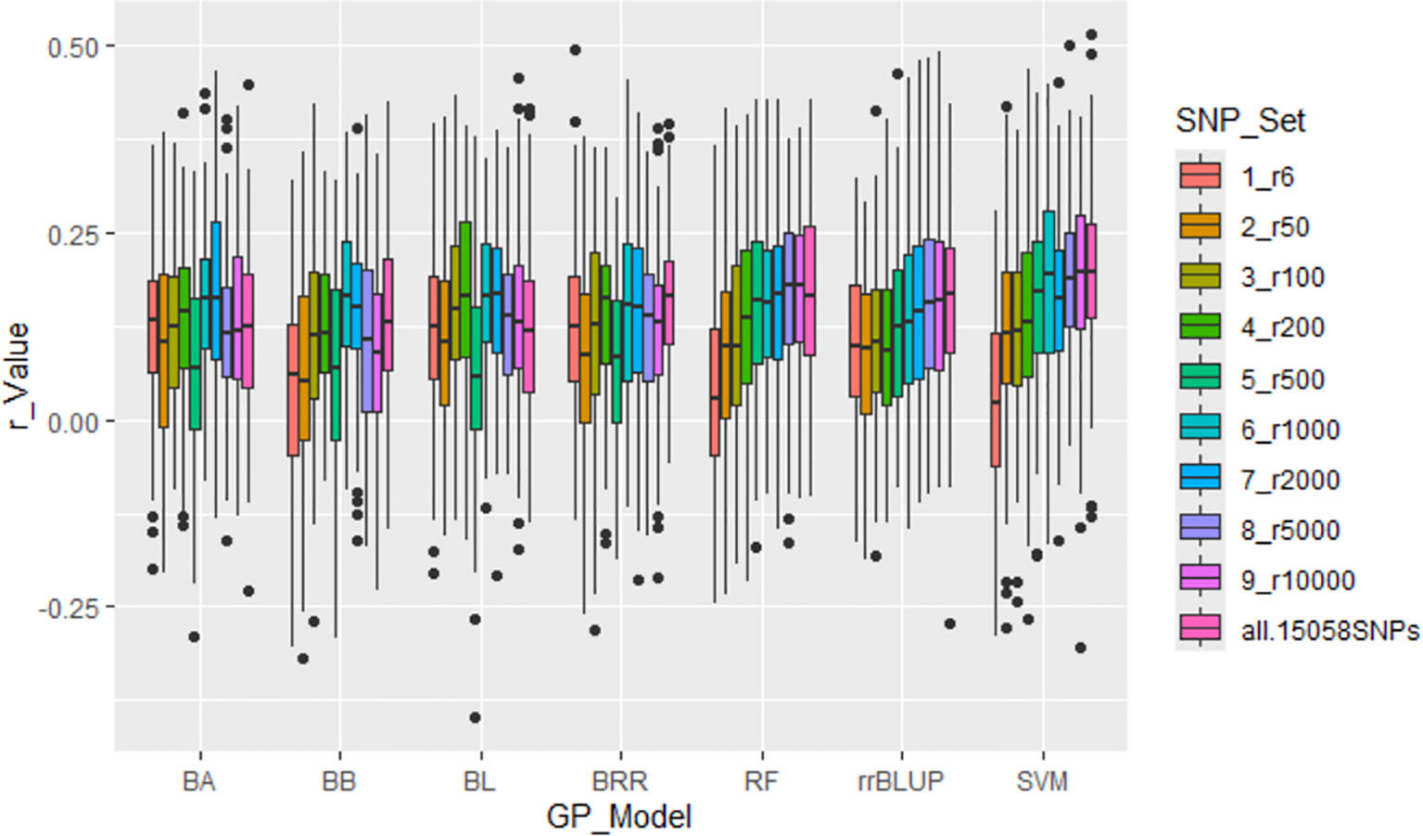
Genomic prediction (r-value) for the tallness trait in 307 spinach accessions using ten different SNP sets, ranging from 6 to 15,058 randomly selected SNPs, in cross-prediction. Prediction accuracy was estimated using seven models: BA, BB, BL, BRR, RF, rrBLUP, and SVM.

All seven GP models—BA, BB, BL, BRR, rrBLUP, RF, and SVM—showed similar r-values across SNP sets, ranging from r6 to all.15058SNPs, with r-values averaging from 0.08 (r6) to 0.15 (all.15058SNPs). These results demonstrated that r-values increased as more SNPs were used (**Supplementary Table S3**; **Figure 5**; **Supplementary Figure S7**). However, the overall prediction accuracy remained low, as indicated by these r-values.

### GP by GWAS-derived SNP markers

#### GWAS-derived SNP markers from whole panel and self-prediction

**Figure 6:**
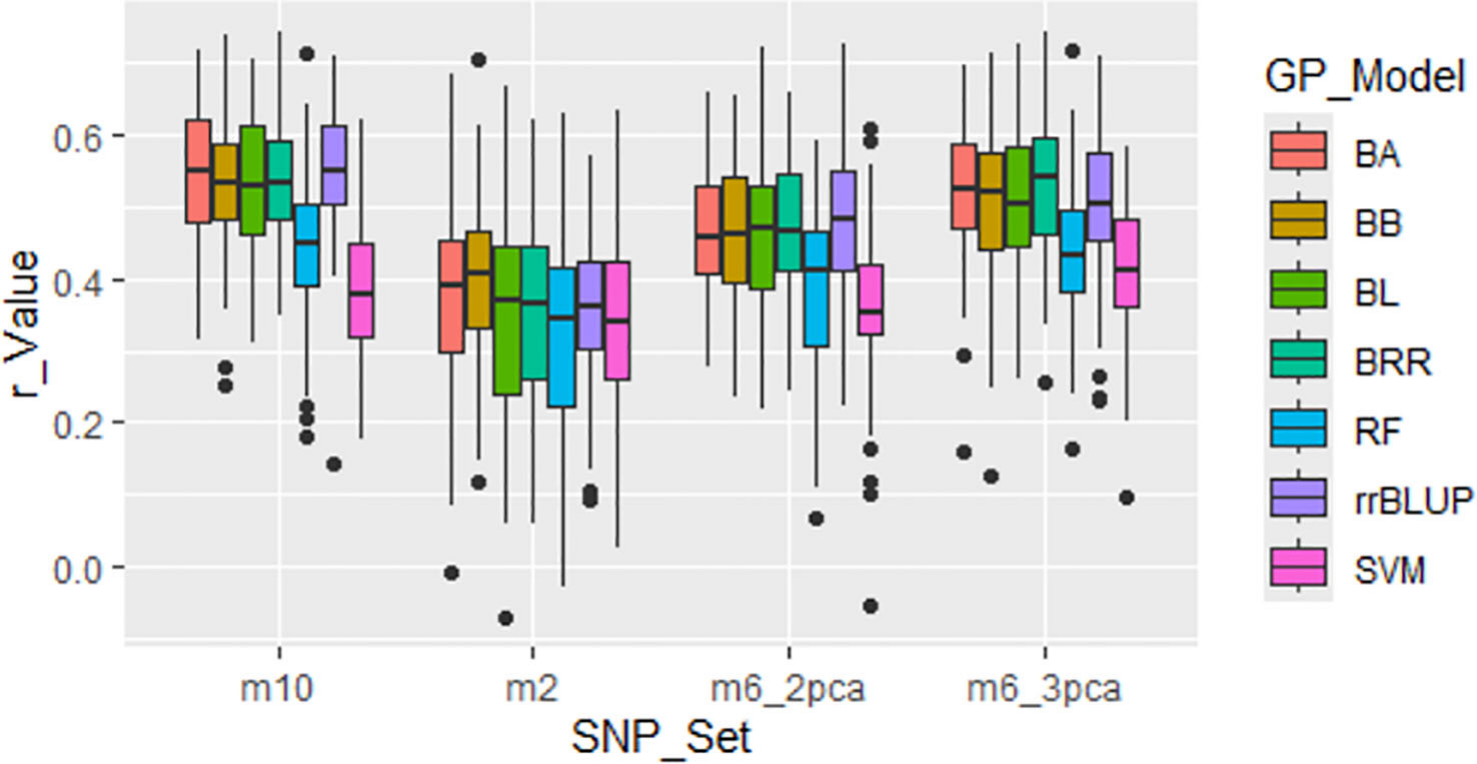
Genomic prediction (r-value) of four GWAS-derived SNP marker sets (m10, 10 markers; m2, 2 markers; m6_2pca, 6 markers with PCA=2; m6_3pca, 6 markers with PCA=3). Prediction was conducted through cross-population analysis with five-fold cross-validation (training:validation = 4:1) using seven genomic prediction (GP) models: BA, BB, BL, BRR, rrBLUP, RF, and SVM.

Four GWAS-derived SNP sets were evaluated: m2 (2 markers), m6_2pca (6 markers with PCA=2), m6_3pca (6 markers with PCA=3), and m10 (10 markers). These sets showed relatively high r-values (**Figure 6**; **Supplementary Table S3**), with average r-values of 0.36, 0.44, 0.49, and 0.50 for m2, m6_2pca, m6_3pca, and m10, respectively, thereby validating their association with the tallness trait within the panel. However, these r-values are expected to decline when the markers are applied in across-population predictions.

#### GWAS-derived SNP markers from 80% of the whole panel

**Figure 7:**
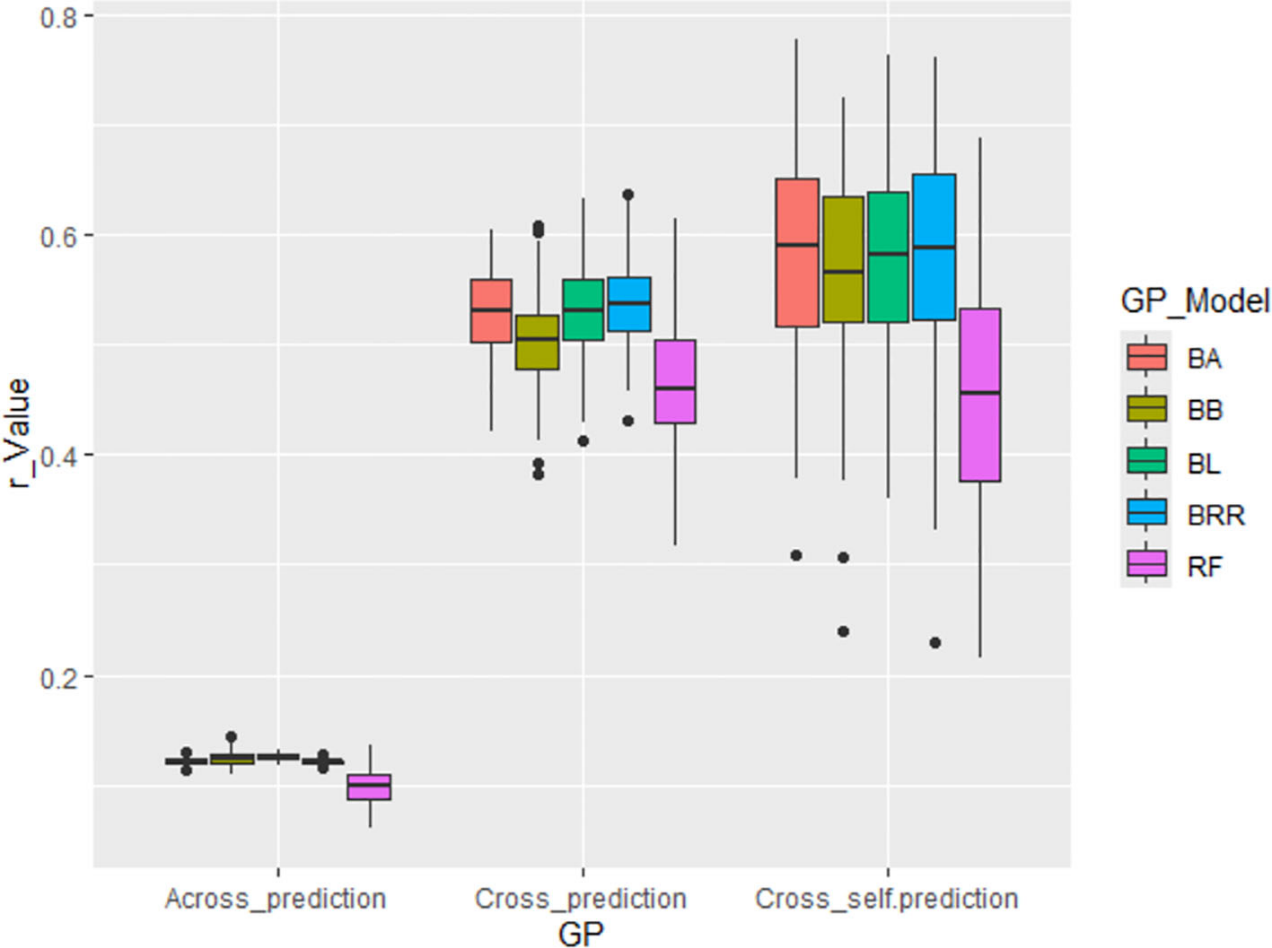
Genomic prediction (GP) accuracy (r-value) for tallness using GWAS-derived SNP markers. Three prediction strategies were applied: (i) Across_prediction – Using GWAS-derived SNP markers from the training set (80% of the population, 246 accessions) to predict the validation set (20%, 61 accessions); (ii) Cross_prediction – Using all associated SNP markers to predict the entire population (307 accessions); and (iii) Cross_self_prediction – Using GWAS-derived SNP markers from the training set (80%) to predict the training set itself.

Across all scenarios, GWAS-derived SNP markers from 80% of the whole panel generally produced moderate prediction accuracies, with an average r-value of 0.51, ranging from 0.47 in the RF model to 0.54 in the BRR model in cross-population predictions. In cross-self-population predictions, the average r-value increased to 0.55, ranging from 0.46 in RF to 0.58 in BA, BL, and BRR. However, prediction accuracy dropped significantly in across-population predictions, with an average r-value of only 0.12, ranging from 0.10 in RF to 0.12 in the other four Bayesian models (**Supplementary Table S4**; **Figure 7**). These findings confirm that the GWAS-derived SNP markers are associated with the tallness trait, but they do not support the application of GS for improving tallness in spinach breeding programs, primarily due to the low predictive ability observed in across-population predictions (**Supplementary Table S4**; **Figure 7**).

#### GWAS-derived SNP markers using GAGBLUP in GAPIT3

**Figure 8:**
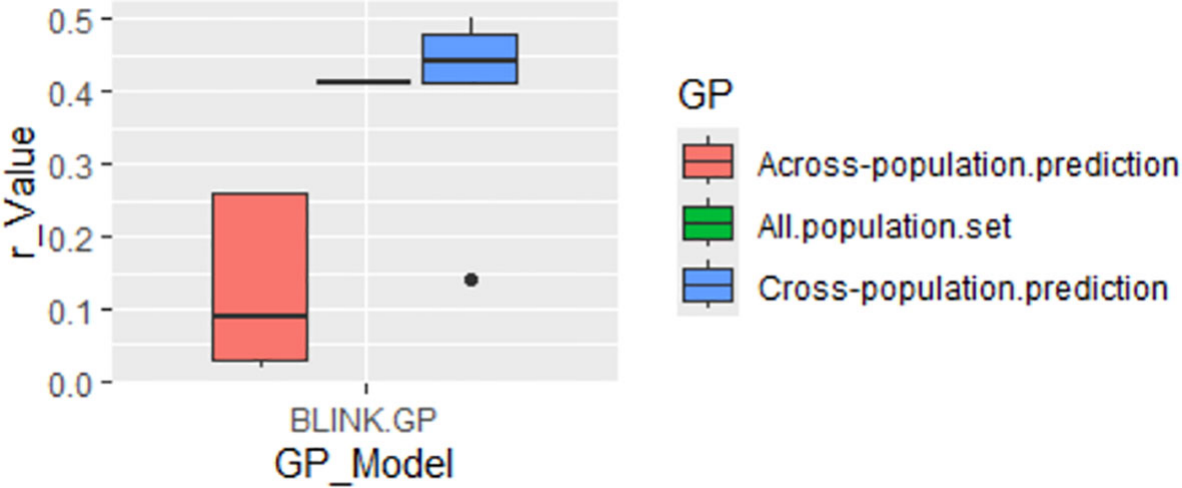
Genomic prediction (GP) (r-value) for tallness using the GAGBLUP (BLINK) model in GAPIT3.

GP was conducted using the GAGBLUP (BLINK) model in GAPIT3 (**Figure 8**). The reference prediction (self-prediction = All.population.set) and cross-population prediction yielded r-values of 0.41 and 0.39, respectively (**Figure 8**). However, the r-value dropped significantly to 0.13 in across-population predictions. These findings suggest that GP using only the significant SNP markers identified by GAGBLUP may not be highly effective for selecting the tallness trait in spinach through GS across populations.

### Genetic prediction using difference genomic models

Building on the GWAS-derived SNP marker sets, we further evaluated prediction accuracy using seven GP models (BA, BB, BL, BRR, rrBLUP, RF, and SVM) under both cross- and across-population analyses. Overall, all models exhibited comparable r-values (**Supplementary Tables S3**, **S4**; **Figures 5**–**7**; **Supplementary Figure S7**), with some variation depending on the marker set.

- For the ten randomly selected SNP sets, all models yielded average r-values of 0.11 or 0.12 (**Supplementary Table S3**; **Figure 5**; **Supplementary Figure S7**).
- For the four GWAS-derived SNP sets (m10: 10 markers; m2: 2 markers; m6_2pca: 6 markers with PCA=2; m6_3pca: 6 markers with PCA=3), BA, BB, BL, BRR, and rrBLUP produced similar average r-values ranging from 0.46 to 0.48, whereas RF and SVM were slightly lower, with 0.44 and 0.38, respectively (**Supplementary Table S3**; **Figure 6**).

These results indicate that rrBLUP and the four Bayesian models (BA, BB, BL, and BRR) are particularly well-suited for predicting tallness in spinach and are recommended for GS of this trait in spinach molecular breeding programs.

## Discussion

### Phenotyping of tallness

The 307 spinach accessions exhibited significant phenotypic variation in tallness, highlighting the complexity and quantitative nature of this trait. In addition, the observed range, spanning 4.5cm to 16.2cm, indicates a broad genetic base, which is essential for successful GWAS and breeding programs aimed at improving plant height. This diversity is consistent with findings in other crops, such as rice and maize, where height is influenced by multiple genes with small effects (Huang and Han, 2016; Yu and Buckler, 2006). Polygenic traits often result in continuous phenotypic variation, which is exactly what we observed in this spinach population.

The identification of particularly tall accessions, such as PI445784 and PI192945, suggests the presence of favorable alleles in these accessions that could be valuable in breeding programs. This finding aligns with studies on wheat and barley, where specific alleles have been identified and exploited to successfully enhance plant height (Chitwood et al., 2016).

The coefficient of variation of 21.3% further indicates substantial phenotypic variability, which is beneficial for selection and increases the likelihood of detecting significant genetic associations. Similar levels of variability have proven advantageous in other crops, supporting the use of diverse panels in GWAS to identify key loci associated with target traits (Magar et al., 2021). Thus, the observation of substantial variability in this study confirms the suitability of this spinach panel for uncovering the genetic underpinnings of tallness and paves the way for more effective breeding strategies.

### PCA and phylogenetic analysis

The population structure and genetic diversity of spinach have been extensively explored using various methodologies, including SNP markers and phylogenetic analyses (Shi et al., 2016). Spinach exhibits significant variability in key traits essential for breeding and crop improvement, such as plant height and leaf morphology (Rashid et al., 2020b). In this study, we utilized high-density SNP data and PCA to assess the genetic diversity of 307 spinach accessions. The results revealed three distinct sub-populations, consistent with earlier studies that highlighted the complex genetic structure of spinach germplasm (Patterson et al., 2006; Saitou and Nei, 1987).

### Association study

In the association study on spinach tallness, which utilized multiple models (GLM, MLM, MLMM, FarmCPU, and BLINK) within the GAPIT3 framework, we detected consistent deviations in the QQ plots, suggesting that the identified SNPs likely contribute to the observed phenotypic variation in height. This finding echoes previous research on other crops, such as rice, maize, and wheat, where plant height has been demonstrated as a complex polygenic trait influenced by multiple loci (Huang and Han, 2016; Yu and Buckler, 2006). The identification of significant SNPs in these crops has been crucial not only for understanding the genetic basis of height but also for guiding breeding programs aimed at improving this trait. Specifically, certain alleles have been exploited to enhance barley stature, further illustrating the value of SNP identification for crop improvement (Chitwood et al., 2016).

The present GWAS identified ten SNPs that exceeded the significance threshold, making them suitable candidates for marker-assisted selection. Targeted breeding based on genetic markers has been successfully applied in crops such as maize, rice, and wheat to develop superior cultivars with improved yield and adaptability (Collard and Mackill, 2008; Huang and Han, 2016; Yu and Buckler, 2006).

### Candidate gene identification/detection

In this study, nine candidate genes were identified within the specific LD decay regions corresponding to eight of the ten associated SNP markers for the tallness trait in spinach (**Table 1** &), suggesting these genes may play important roles in controlling plant height. Both *SOV4g016060* (U6 snRNA-associated Sm-like protein LSm5), located near SOVchr4_38323167 on chromosome 4, and *SOV5g028680* (cleavage and polyadenylation specificity factor subunit 2), near SOVchr5_70192260 on chromosome 5, are involved in Ribonucleic acid (RNA) processing—a critical function previously linked to growth regulation in multiple crops. For instance, in maize, genes involved in RNA processing have been shown to influence plant height by regulating the expression of growth-related genes (Huang and Han, 2016). Similarly, in rice, RNA processing genes can affect both height and yield (Gong et al., 2021). In barley, genes associated with RNA processing have been found to regulate flowering time and overall plant stature (Nitcher et al., 2013).

Two additional candidate genes on chromosome 4, *SOV4g059190* (outer envelope membrane protein 7-like) and *SOV4g059200* (epimerase domain-containing protein), both located near SOVchr4_188084338, are associated with metabolic and transport processes. These processes are crucial for cell elongation and biomass accumulation, as previously evidenced in rice and maize. In rice, genes related to metabolic pathways have been linked to the regulation of internode elongation, a key factor in determining plant height (Yu and Buckler, 2006). In maize, the transport of nutrients and growth regulators is critical for the development of tall plants (Hütsch and Schubert, 2018).

The candidate gene identified in this study, *SOV6g020520* (LETM1 and EF-hand domain-containing protein 1 mitochondrial), located near SOVchr6_91175684 on chromosome 6, suggests a role for mitochondrial function in regulating spinach height. Mitochondria are essential for energy production, which is necessary to sustain the metabolic demands of growing plants. Studies in wheat and barley have demonstrated that mitochondrial function is closely linked to plant vigor and height, with efficient energy production supporting taller growth (Lozano et al., 2009).

### Genomic prediction for genomic selection of tallness trait

Integration of GP models into breeding programs has become an essential tool for enhancing crop traits, such as plant height, through GS. In this study, we evaluated the performance of seven GP models in predicting tallness in 307 spinach accessions using both randomly selected SNP sets and GWAS-derived SNP marker sets.

The seven GP models—BA, BB, BL, BRR, rrBLUP, RF, and SVM—showed similar r-values across SNP sets from r6 to all.15058SNPs, averaging from 0.08 (r6) to 0.16 (r1000) (**Supplementary Table S3**, **Figure 5**, **Supplementary Figure S7**). The r-value generally increased as the number of SNPs in the set increased, but the improvement plateaued after 1,000 SNPs. The results demonstrated that increasing the number of SNPs from six to 15,058 led to a progressive rise in prediction accuracy (r-value), stabilizing around 1,000 SNPs across all models. These findings underscore the necessity of utilizing a sufficient number of markers to achieve reliable predictions, consistent with previous research emphasizing the importance of genome-wide coverage for accurate GP (Heslot et al., 2012). However, all r-values were low, indicating that GP may not be efficient for predicting the tallness trait.

Despite the general trend of larger SNP sets yielding higher prediction accuracy, the four GWAS-derived SNP sets—m2, m6_2pca, m6_3pca, and m10—achieved relatively high average r-values of 0.36, 0.44, 0.49, and 0.50, respectively (**Supplementary Table S3**; **Figure 6**). Notably, even the two-SNP marker set (m2) produced a relatively high average r-value of 0.36 across the seven models. These findings indicate that a small number of strategically selected SNPs can provide substantial predictive power, particularly when the markers are tightly linked to the trait of interest (Zhao et al., 2021). Similar results have been reported in other crops, where GWAS-derived markers significantly enhanced prediction models for complex traits (Minamikawa et al., 2021).

Among the seven GP models evaluated, rrBLUP and the four Bayesian GS models (BA, BB, BL, and BRR) produced higher r-values when using GWAS-derived SNP marker sets. The superior performance of rrBLUP may be attributed to its strong capacity to capture additive genetic variance, which is particularly important for polygenic traits such as plant height, where numerous genes with small effects collectively contribute to phenotypic variation (Goddard and Hayes, 2007; Crossa et al., 2017). By contrast, the Bayesian GS models exhibited less consistent trends in prediction ability as marker numbers increased, compared with the other models (**Figure 5**). This variability may reflect the influence of model-specific assumptions and prior distributions inherent in Bayesian frameworks, which may interact differently with varying SNP densities. Further investigation will be required to clarify these dynamics.

## Conclusion

Phenotypic evaluation revealed substantial variability in plant height, with seven accessions exhibiting exceptional tallness identified as promising candidates for breeding. Ten SNPs on chromosomes 1, 2, 4, 5, and 6 were strongly associated with tallness, with particularly notable contributions from markers on chromosome 6. Within LD decay regions, nine candidate genes related to F-box domain-containing proteins, RNA processing, metabolic pathways, and mitochondrial function were identified, providing valuable targets for further functional characterization. Genomic prediction analyses demonstrated that rrBLUP, in particular, achieved high predictive accuracy, even when using a small GWAS-derived SNP set. This highlights the potential of these markers for forecasting genetic potential for plant height. Collectively, these findings provide breeders with valuable molecular tools to facilitate targeted selection and genotyping, supporting the development of spinach varieties optimized for mechanical harvesting and market preferences. By integrating genomic insights with conventional breeding approaches, this study lays a foundation for sustainable and economically viable strategies to improve spinach height.

## Data Availability

The datasets presented in this study can be found in online repositories. https://doi.org/10.6084/m9.figshare.28603517.v1 The names of the repository/repositories and accession number(s) can be found in the article/**Supplementary Material**.
